# Day-case success or why still in hospital after total hip, total knee, and medial unicompartmental knee arthroplasties? a prospective multicentre cohort study on 6,142 patients from a public healthcare system

**DOI:** 10.1302/2633-1462.511.BJO-2024-0125.R1

**Published:** 2024-11-05

**Authors:** Oddrún Danielsen, Christian B. Jensen, Claus Varnum, Thomas Jakobsen, Mikkel R. Andersen, Manuel J. Bieder, Søren Overgaard, Christoffer C. Jørgensen, Henrik Kehlet, Kirill Gromov, Martin Lindberg-Larsen

**Affiliations:** 1 Center for Fast-track Hip and Knee Replacement, Copenhagen, Denmark; 2 Department of Orthopaedic Surgery and Traumatology, Odense University Hospital and Svendborg, Svendborg, Denmark; 3 Department of Orthopaedic Surgery, Hvidovre University Hospital, Hvidovre, Denmark; 4 Department of Orthopaedic Surgery, Lillebaelt Hospital – Vejle, Vejle, Denmark; 5 Department of Orthopaedic Surgery, Aalborg University Hospital, Aalborg, Denmark; 6 Department of Orthopaedic Surgery, Copenhagen University Hospital, Herlev-Gentofte, Copenhagen, Denmark; 7 Department of Orthopaedic Surgery, Næstved, Slagelse and Ringsted Hospitals, Slagelse, Denmark; 8 Department of Orthopaedic Surgery and Traumatology, Copenhagen University Hospital, Bispebjerg, Copenhagen, Denmark; 9 Department of Clinical Medicine, Faculty of Health and Medical Sciences, University of Copenhagen, Copenhagen, Denmark; 10 Department of Anaesthesia, Hospital of Northern Zeeland, Hillerød, Denmark; 11 Section of Surgical Pathophysiology, Copenhagen University Hospital, Rigshospitalet, Copenhagen, Denmark

**Keywords:** Arthroplasty, Hip arthroplasty, Knee arthroplasty, Day-case surgery, Fast-track, medial unicompartmental knee arthroplasties, Hip, Total hip and total knee arthroplasty, hip and knee arthroplasties, spinal anaesthesia, hip and knee arthroplasties, knee, primary total hip arthroplasty, cohort study, Total hip and total knee arthroplasty

## Abstract

**Aims:**

Day-case success rates after primary total hip arthroplasty (THA), total knee arthroplasty (TKA), and medial unicompartmental knee arthroplasty (mUKA) may vary, and detailed data are needed on causes of not being discharged. The aim of this study was to analyze the association between surgical procedure type and successful day-case surgery, and to analyze causes of not being discharged on the day of surgery when eligible and scheduled for day-case THA, TKA, and mUKA.

**Methods:**

A multicentre, prospective consecutive cohort study was carried out from September 2022 to August 2023. Patients were screened for day-case eligibility using well defined inclusion and exclusion criteria, and discharged when fulfilling predetermined discharge criteria. Day-case eligible patients were scheduled for surgery with intended start of surgery before 1.00 pm.

**Results:**

Of 6,142 primary hip and knee arthroplasties, eligibility rates for day-case surgery were 34% for THA (95% CI 32% to 36%), 34% for TKA (95% CI 32% to 36%), and 52% for mUKA (95% CI 49% to 55%). Surgery before 1.00 pm was achieved in 85% of eligible patients. The day-case success rate among patients with surgery before 1.00 pm was 59% (95% CI 55% to 62%) for THA, 61% (95% CI 57% to 65%) for TKA, and 72% (95% CI 68% to 76%) for mUKA. Overall day-case success rates (eligible and non-eligible) were 19% (95% CI 17% to 20%) for THA, 20% (95% CI 18% to 21%) for TKA, and 42% (95% CI 39% to 45%) for mUKA. Adjusted analysis confirmed higher day-case success in eligible mUKA patients (odds ratio 1.9 (1.6 to 2.3)) compared to TKA and THA patients. Primary causes for day-case failure were mobilization issues (9% to 12% between procedures), prolonged spinal anaesthesia (4% to 9%), and postoperative nausea and vomiting (PONV) (4% to 14%).

**Conclusion:**

THA and TKA patients showed comparable eligibility (34%) with similar day-case success rates (59 to 61%), whereas mUKA patients demonstrated higher eligibility (52%) and day-case success (72%). Mobilization issues, prolonged spinal anaesthesia, and PONV were the most frequent causes for not being discharged.

## Introduction

The feasibility of discharging selected patients on the day of surgery after hip and knee arthroplasty has been reported in previous studies.^1,2^ Discharge on day of surgery is used increasingly due to the potential for reduced bed occupancy and costs. As this trend is evolving and becomes more prevalent, it is important to reflect on the existing data and protocols to clarify which patients are best suited for discharge on the day of surgery. Eligibility criteria for day-case surgery based on clinical assessment and comorbidity considerations are debatable.^3,4^

The role of surgical procedure type for eligibility is less well known, but the use of day-case procedures has been reported higher after medial unicompartmental knee arthroplasty (mUKA) than after primary total hip arthroplasty (THA) and total knee arthroplasty (TKA) on a nationwide basis.^5,6^ However, limited data exist on differences in day-case success rates between surgical procedures in patients with similar comorbidity profiles fulfilling identical eligibility criteria. Furthermore, a better understanding of the causes of not being discharged on the day of surgery despite being found eligible is important for further improvement.

The purpose of this study was to analyze the effect of the surgical procedure on successful discharge on day of surgery. Furthermore, to analyze causes of not being discharged on day of surgery when found eligible and scheduled for day-case THA, TKA, and mUKA.

## Methods

This was a prospective consecutive multicentre cohort study conducted in accordance with RECORD guidelines for the reporting of routinely collected, observational data.^7^

### Setting

The centres collaborating within the Centre for Fast-track Hip and Knee Replacement consist of seven public arthroplasty centres distributed across all regions in Denmark, accounting for approximately 40% of the total annual nationwide procedures in this field.^8^

### Study population

Consecutive patients undergoing primary THA, TKA, and mUKA from 1 September 2022 to 31 August 2023 from seven public departments of orthopaedic surgery were included. Patients were screened for day-case eligibility using well-defined inclusion and exclusion criteria (Table I) and discharged when they fulfilled predetermined discharge criteria (Table II). Patients eligible for discharge on day of surgery were scheduled with an intended start of surgery before 1.00 p.m. The protocol and implementation of day-case surgery in our collaboration has been published.^9,10^

**Table I.** Inclusion and exclusion eligibility criteria for planned discharge on day of surgery.

Inclusion criteria
Unilateral elective primary THA, TKA, or UKA
Aged 18 to 80 years

Exclusion criteria
Acute myocardial infarction, cerebrovascular accident, transient ischaemic attack, or coronary atherosclerotic disease within last 3 months
Unstable ischaemic heart disease
Ejection fraction < 40%
Glomerular filtration rate < 60 ml/min/1.73 m^2^
Chronic obstructive pulmonary disease with home oxygen
Insulin-dependent diabetes mellitus
Sleep apnoea requiring mechanical treatment
Clinical Frailty Scale ≥ 4
Two or more falls within last 3 months
BMI < 18.5 kg/m^2^ or > 40 kg/m^2^
Not interested in discharge on day of surgery
No adult present at home during the initial postoperative night*

* This criterion was inadvertently omitted from the protocol paper, but has consistently been applied across all centres.

THA, total hip arthroplasty; TKA, total knee arthroplasty; UKA, unicompartmental knee arthroplasty.

**Table II.** Criteria for discharge on day of surgery.

Activity level
Steady gait with crutches
No dizziness during mobilization
Can use stairs, if required by participant’s home environment

Postoperative nausea and vomiting
Minimal and efficiently treated with or without medication Early Warning System* < 2
Patients with Early Warning System* ≥ 2 must be discussed with a doctor prior to discharge

Pain
Numeral Rating Scale† < 3 at rest
Numeral Rating Scale† < 5 when walking 5 metres
Or otherwise, acceptable level of pain assessed by the participant, regardless of numeral rating scale score

Postoperative bleeding
Should be consistent with expected blood loss for this procedure and not require repeated dressing change

* System based on NEWS2 from the Royal College of Physicians.^11^

† Based on a scale of 0 to 10, with 0 being no pain and 10 being the worst pain imaginable.

### Data sources

Preoperative patient-reported data and data from patient files were prospectively recorded and securely stored in an online REDCap database^12^ provided by the Open Patient Data Explorative Network (OPEN) at Odense University Hospital.^9^ Additionally, eligibility and reasons for patients not being eligible were prospectively registered through dedicated research staff at individual hospitals with physician back-up if necessary. Reasons for not being discharged on the day of surgery were prospectively recorded and classified based on unmet discharge criteria.

### Variables

Data on the number of hip and knee arthroplasties performed were available from the REDCap database. The primary outcome was the proportion of day-case patients according to surgical procedure type in eligible patients scheduled for day-case surgery (surgery before 1.00 pm), and overall. A day-case patient was defined as a patient who was admitted on the day of surgery and discharged on the same day.

The secondary outcome was proportions of causes for not being discharged, despite being eligible and scheduled for day-case surgery.

### Quantitative variables

Causes for patients not being discharged on day of surgery, despite being eligible and scheduled for day-case surgery, were registered by the research staff in accordance to the protocol.^9^ A designated category was assigned as “other” when specified causes were inadequate. A checkmark was placed next to all causes leading to prolonged hospitalization, hence the possibility of patients having more than one cause.

The variable “late return to ward” refers to patients returning to the ward too late in the afternoon, providing the staff with no time to prepare the patient for discharge according to the discharge criteria. The variable “mobilization issue” covers all causes of the patient not being mobilized sufficiently for discharge, including both physical (e.g. motor blockade, pain, dizziness) and logistical factors, such as the absence of a physiotherapist to give instruction in rehabilitation exercises before discharge. The variable “surgical complications” includes causes related to the operative intervention that could not be classified as expected according to the specific procedure, e.g. significant bleeding or an exceptionally long operation.

### Statistical analysis

Continuous variables were presented as means with SD, while categorical variables were described using absolute and relative frequencies. Percentages of categorical variables were presented with 95% CIs. The association between surgical procedure and successful discharge on day of surgery was assessed with a multivariate logistic regression model, adjusted for the continuous variables of age and BMI, and the categorical variables sex, Clinical Frailty Scale (CFS),^13^ and centre. The results are presented as odd ratios (ORs) with 95% CI. Data were analyzed using Stata Statistical Software v. 18 (StataCorp, USA).

### Ethics

Patients were included after informed consent. The management of eligible patients for day-case surgery adhered to the standard of care outlined in the protocol at the participating centres,^9^ obviating the necessity for ethical approval. The study was preregistered at ClinicalTrials.gov (NCT05613439) and in the region of Southern Denmark, with approval granted for data processing (Journal No 22/39454).

## Results

**Fig. 1:**
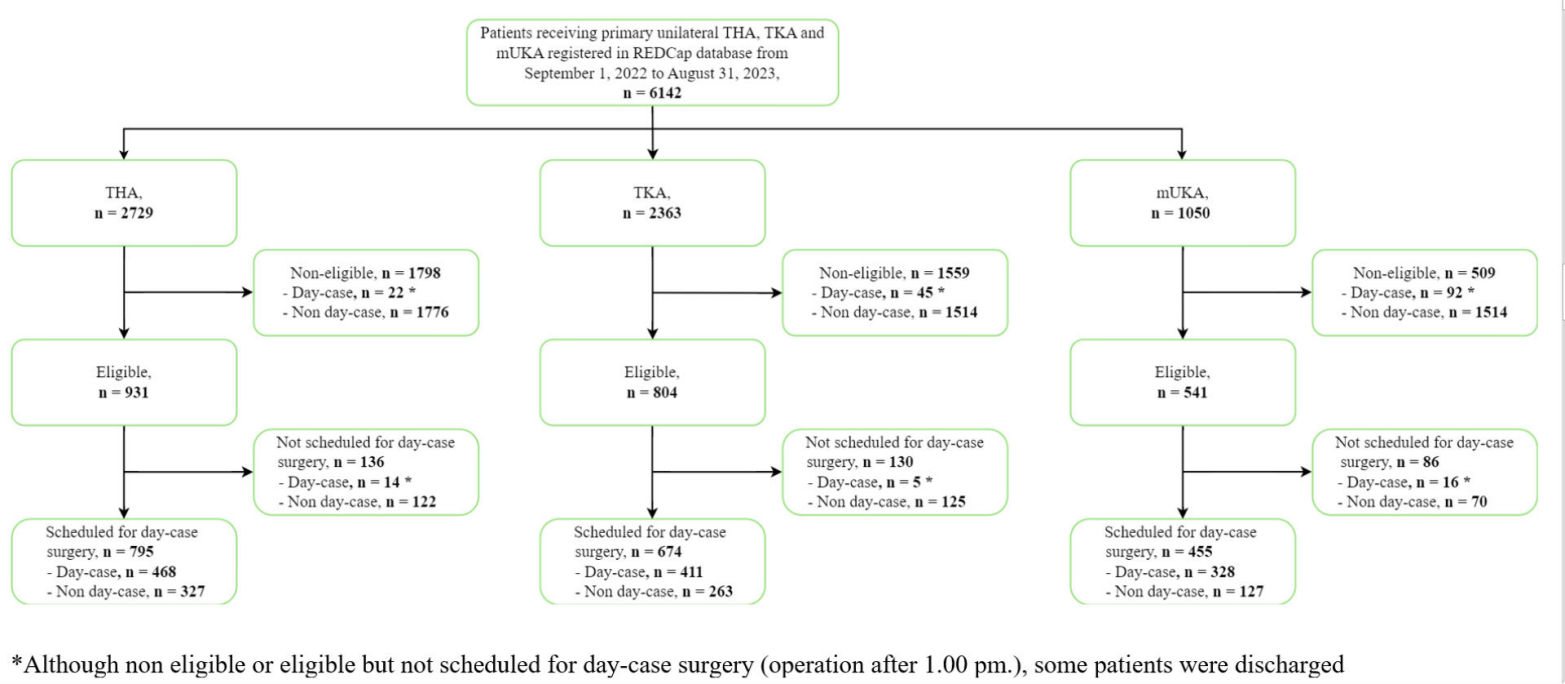
Flowchart of patient inclusion in the study. mUKA, medial unicompartmental knee arthroplasty; THA, total hip arthroplasty; TKA, total knee arthroplasty.

Overall, 6,142 patients operated for primary hip and knee arthroplasties were registered in the database in the inclusion period: 2,729 THAs, 2,363 TKAs, and 1,050 mUKAs (31% of all knee arthroplasties) (Figure 1). Baseline characteristics for day-case eligible patients were comparable across all three arthroplasty groups (Table III).

**Table III.** Patient demographic data.

Variable	THA		TKA		mUKA	
	Overall (n = 2,729)	Day-case eligible and scheduled for day-case surgery (n = 795)*	Overall (n = 2,363)	Day-case eligible and scheduled for day-case surgery (n = 411)*	Overall (n = 1,050)	Day-case eligible and scheduled for day-case surgery (n = 328)*
**Mean age**, yrs (SD)	70.3 (10.6)	65.2 (9.9)	70.1 (9.3)	66.3 (8.2)	68.3 (9.3)	65.9 (8.5)
**Sex, %**						
Female	56.7	49.5	59.8	49.5	48.7	41.3
Male	43.4	50.5	40.2	50.5	51.3	58.7
**Mean BMI**, kg/m^2^ (SD)	27.4 (5.2)	27.1 (4.4)	29.4 (5.9)	28.9 (4.7)	29.4 (5.3)	29.2 (4.7)
**Mean CFS** (SD)	3.0 (1.2)	2,2 (0.7)	2.6 (1.2)	2.2 (0.7)	2.6 (1.0)	2.1 (0.8)

* Operation started before 1.00 pm.

CFS, Clinical Frailty Scale; mUKA, medial unicompartmental knee arthroplasty; THA, total hip arthroplasty; TKA, total knee arthroplasty.

**Fig. 2:**
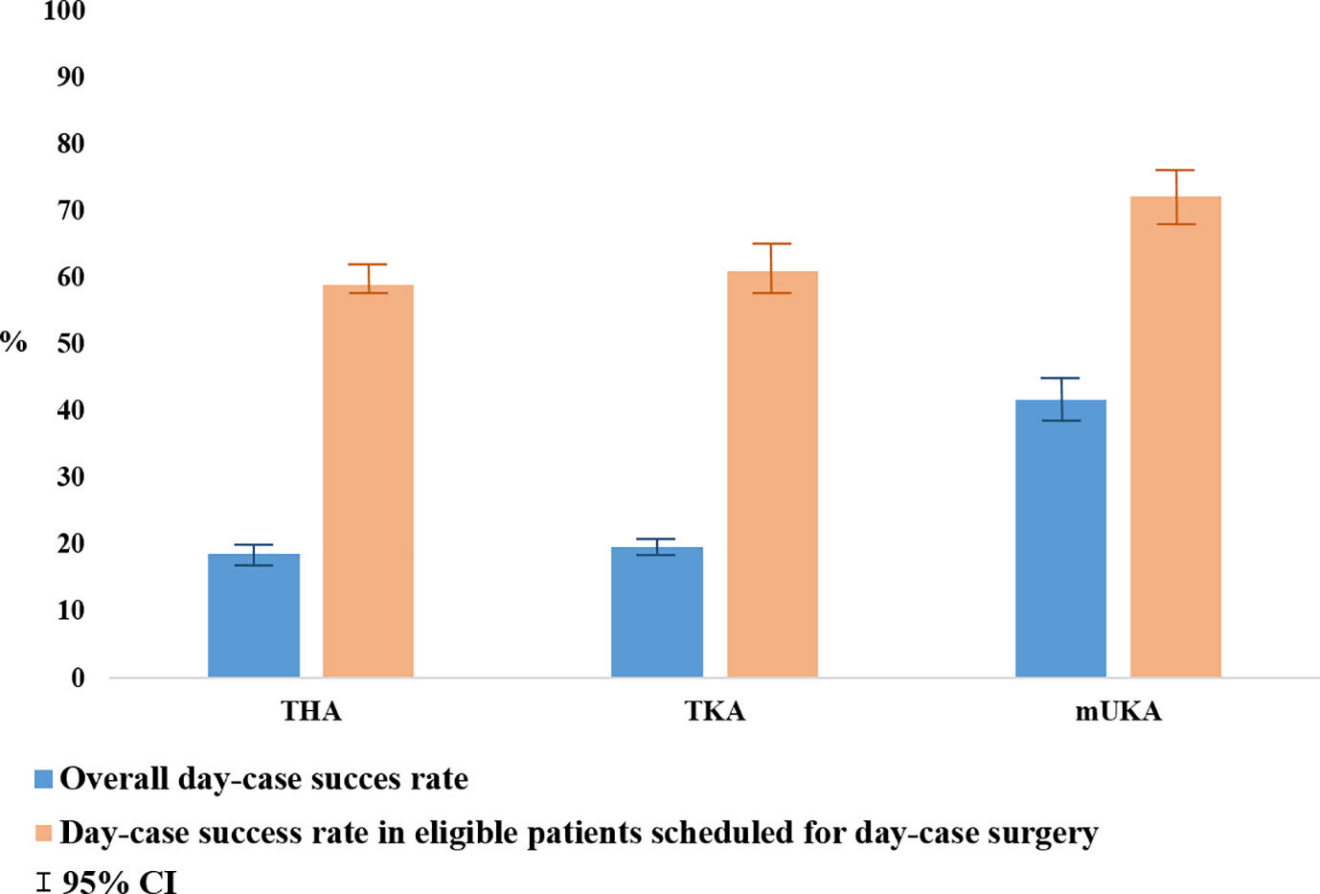
Overall day-case success rates, and day-case success rates in eligible patients scheduled for day-case total hip arthroplasty (THA), total knee arthroplasty (TKA), and medial unicompartmental knee arthroplasty (mUKA) surgery.

The day-case eligibility rates were 34% for THA (95% CI 32% to 36%), 34% for TKA (95% CI 32% to 36%), and 52% for mUKA (95% CI 49% to 55%). Surgery before 1.00 pm was achieved in 85% of eligible patients. The day-case success rate among patients with surgery before 1.00 pm was 59% (95% CI 55% to 62%) for THA, 61% (95% CI 57% to 65%) for TKA, and 72% (95% CI 68% to 76%) for mUKA. Overall day-case success rates (eligible and non-eligible) were 19% (95% CI 17% to 20%) for THA, 20% (95% CI 18% to 21%) for TKA, and 42% (95% CI 39% to 45%) for mUKA (Figure 2). THA and TKA exhibited similar day-case success among eligible patients scheduled for day-case surgery in the adjusted regression analysis. In contrast, the mUKA procedure was associated with a higher rate of day-case success (OR 1.9 (1.6 to 2.3) in the adjusted analysis) (Table IV).

**Table IV.** Logistic regression analysis estimating odds ratios of successful procedure for the day-case eligible patients scheduled for day-case surgery based on surgical procedure type.

Variable	Successful day-case procedure, %	OR (95% CI)	Adjusted OR (95% CI)*
THA (n = 2,729)	51.8	Ref	Ref
TKA (n = 2,363)	51.7	1.1 (0.9 to 1.4)	1.1 (0.9 to 1.4)
mUKA (n = 1,050)	63.6	1.7 (1.3 to 2.2)	1.9 (1.6 to 2.3)

* Adjusted for age, BMI, sex, Clinical Frailty Scale, and centre.

mUKA, medial unicompartmental knee arthroplasty; OR, odds ratio; THA, total hip arthroplasty; TKA, total knee arthroplasty.

**Fig. 3:**
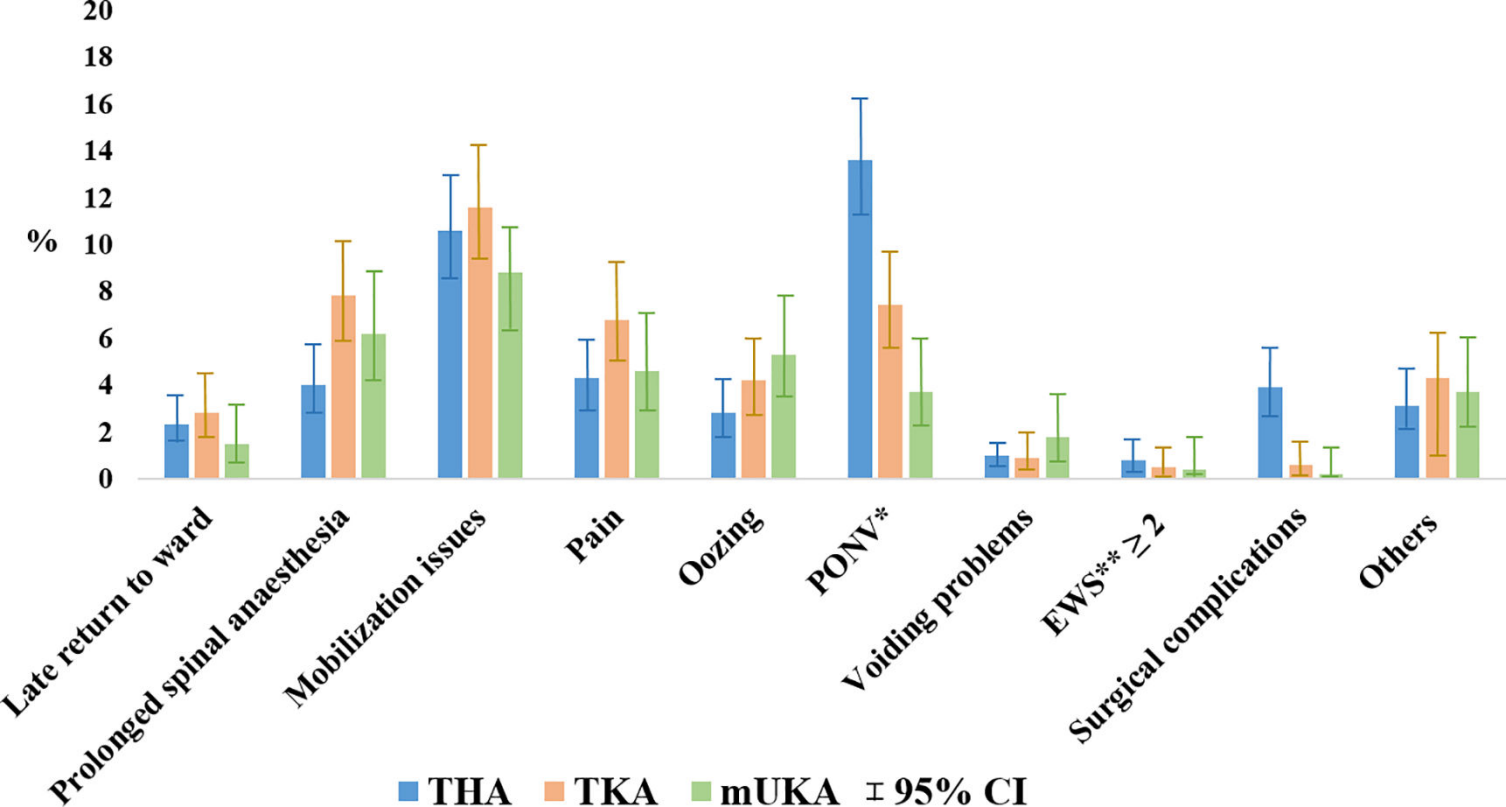
Causes for patients not being discharged on day of surgery despite being eligible and scheduled for day-case surgery according to surgical procedure type. A checkmark was placed next to all causes leading to prolonged hospitalization, hence the possibility of patients having more than one cause. EWS, Early Warning Score; mUKA, medial unicompartmental knee arthroplasty; PONV, postoperative nausea and vomiting; THA, total hip arthroplasty; TKA, total knee arthroplasty.

The primary causes for not being discharged on the day of surgery despite being eligible and scheduled for day-case surgery were mobilization issues: 11% (95% CI 8% to 14%) for THA patients, 12% (95% CI 9% to 15%) for TKA patients and 9% (95% CI 5% to 14%) for mUKA patients; prolonged spinal anaesthesia: 4% (95% CI 3% to 6)% for THA patients, 8% (95% CI 5% to 11%) for TKA patients and 6% (95% CI 3% to 11%) for mUKA patients; and postoperative nausea and vomiting (PONV): 14% (95% CI 11% to 17%) for THA patients, 7% (95% CI 5% to 11%) for TKA patients, and 4% (95% CI 2% to 8%) for mUKA patients (Figure 3).

## Discussion

In this prospective cohort study investigating the impact of surgical procedure on successful discharge on the day of surgery, we found that patients receiving THA and TKA had similar eligibility and overall day-case success rates, while patients receiving mUKA not only had higher eligibility rates but also achieved higher day-case success rates. Furthermore, we found that the primary causes for not being discharged on day of surgery were mobilization issues, prolonged spinal anaesthesia, and PONV.

No significant differences were found in the demographic characteristics of patients undergoing mUKA compared to THA and TKA that could explain the variations in eligibility and day-case success rates between procedures. We believe that the significantly higher rate of day-cases among mUKA patients is explained by the fact that the UKA procedure is a less invasive procedure compared to TKA, hence less pain and faster mobilization could be expected. There also appears to be less orthostatic intolerance after UKA compared to TKA and THA.^14^ Moreover, it may also be attributed to a higher rate of eligible patients, and increased confidence in day-case success after mUKA based on previous experience.^6,15^ Additionally, there might be a tendency to prioritize mUKA patients as the first patients on the operation schedule, thereby enhancing their overall chances of success. However, if we account for these assumptions and only focus on eligible patients with start of surgery before 1.00 pm, mUKA patients still had the highest rate of success (Figure 2). Our findings confirm the advantage of offering mUKA to patients with anteromedial knee arthrosis in order to achieve a higher overall day-case success rate. This may also be reflected in the relatively high frequency (31%) of mUKAs out of all knee arthroplasties performed in our collaboration and in Denmark (27%).^16^

Several studies have shown that discharge on the day of surgery after THA, TKA, and mUKA is possible,^1,2^ but with higher day-case rates for mUKA.^5,17^ However, the set-up and eligibility criteria in these studies vary, hindering sufficient comparison of the day-case success for each surgical procedure. A previous Danish nationwide register study showed that the annual overall proportion of day-cases was 5.4% for THA patients, 2.8% for TKA, and 20% for UKA patients in 2019.^5^ In comparison, we have now increased our overall day-case rates to 20% for both THA and TKA and 40% for mUKA in our multicentre collaboration. This has been achieved by implementation of our protocol and guideline for day-case surgery.^10^

Lack of sufficient mobilization was one of the primary causes of not being discharged on the day of surgery, but we did not differentiate the causes for inadequate mobilization. The relatively high rate of mobilization issues may, therefore, indicate other potential underlying causes, such as logistical issues related to patients not being assessed by a physiotherapist on the day of surgery, or due to prolonged spinal anaesthesia, PONV, pain, or orthostatic intolerance.

Prolonged spinal anaesthesia as an isolated cause preventing discharge on the day of surgery was also relatively prevalent. In our protocol and guideline for day-case surgery, we did not set a requirement for the use of either general anaesthesia or spinal anaesthesia. If using spinal anaesthesia, the recommendation was to focus on using relatively short-acting blockades with smaller volumes or concentrations of local anaesthetic in order to facilitate mobilization within two hours postoperatively.^9^ Nevertheless, prolonged spinal anaesthesia still hindered day-case success, and previous studies have shown that general anaesthesia may result in earlier recovery; less pain, dizziness, and nausea; and earlier recovery of walking ability compared with spinal anaesthesia.^18,19^ Hence, general anaesthesia may be used more frequently to improve day-case success rates, especially if patients are not scheduled as the first in the operating theatre.

Of note, we found a high rate of PONV as a possible cause of not reaching discharge on day of surgery in THA patients compared to TKA and mUKA patients. Previous studies have found PONV as a possible reason for not being discharged on the day of surgery,^20-22^ but to our knowledge, no previous study has found PONV to be more likely in THA patients. Further investigation on this subject is needed.

The strength of this study lies in the well-defined and detailed protocol for the day-case surgery,^9^ enabling other surgical centres to compare their set up and results with ours.

The arthroplasty centres affiliated with the Centre for Fast-track Hip and Knee Arthroplasty are distributed across all regions in Denmark, constituting approximately 40% of the total annual surgeries in the area nationwide. This strategic distribution holds the potential to enhance the generalizability of our findings.

Our study also has possible limitations. There is a small possibility that the causes of not being discharged on day of surgery may be affected by misclassification and information bias. To limit this, possible causes of not being discharged on the day of surgery aligned with the discharge criteria were included in our database to assist research staff when filling in causes of not being discharged.

Within the multicentre collaboration, the perioperative management of day-case patients was optimized and all centres adhered to the same guideline for discharge on the day of surgery.^9^ A relatively high percentage (15%) of eligible patients were undergoing surgery after 1.00 pm, although the study protocol recommends scheduled surgery before this time for all day-case eligible patients.^9^ This underscores the need for improvement, particularly in the logistical setup within the collaboration, and will be a focal point for targeted enhancement.

In conclusion, we found similar day-case eligibility and success for patients undergoing THA and TKA, while mUKA patients had higher rate of eligibility and day-case success. Mobilization issues, prolonged spinal anaesthesia and PONV were the major causes of not being discharged on day of surgery.

**Take home message**

- Total hip and total knee arthroplasty patients showed comparable eligibility (34%) with similar day-case success rates (59% to 61%), whereas medial unicompartmental knee arthroplasty patients demonstrated higher eligibility (52%) and day-case success (72%).

- Mobilization issues, prolonged spinal anaesthesia, and postoperative nausea and vomiting were the most frequent causes for not being discharged.

## Data Availability

The datasets generated and analyzed in the current study are not publicly available due to data protection regulations. Access to data is limited to the researchers who have obtained permission for data processing. Further inquiries can be made to the corresponding author.
